# Biomarkers to Target Heterogeneous Breast Cancer Stem Cells

**DOI:** 10.4172/2155-9929.S8-006

**Published:** 2012

**Authors:** Wendy W. Hwang-Verslues, Wen-Hwa Lee, Eva Y.-H.P. Lee

**Affiliations:** 1 Genomics Research Center, Academia Sinica, No. 128, Sec. 2, Academia Road, Taipei 115, Taiwan; 2 Department of Biological Chemistry, University of California, Irvine, CA 92697, USA

## Abstract

Breast cancer is the most common cancer and the second leading cause of death in U.S. women. Due to early detection and advanced treatment, the breast cancer death rate has been declining since 1990. However, disease recurrence is still the major obstacle in moving from therapy to truly curative treatments. Recent evidence has indicated that breast cancer recurrence is often caused by a subpopulation of breast cancer cells. This subset of cancer cells, usually referred to as breast cancer stem cells (BCSCs), exhibits stem cell phenotypes. They can self-renew and asymmetrically divide to more differentiated cancer cells. These cells are also highly resistant to conventional therapeutic reagents. Therefore, identifying and characterizing these BCSC subpopulations within the larger population of breast cancer cells is essential for developing new strategies to treat breast cancer and prevent recurrence. In this review article, we discuss the current proposed model for the origin of tumor heterogeneity, summarize the recent findings of cell surface and cytoplasmic markers for BCSC identification, review the regulatory mechanisms by which BCSCs maintain or non-cancer stem cells acquire BCSC characteristics, describe the proposed strategies to eliminate BCSCs, and highlight the current limitations and challenges to translate basic BCSC research to clinical application including establishment of clinical biomarkers and therapeutic treatments specifically targeting BCSCs.

## Introduction

### A hybrid model for the origin of tumor heterogeneity

There are two concepts for explaining tumor heterogeneity: the CSC hypothesis and clonal evolution model [1,2]. The CSC hypothesis proposes that CSCs share similar properties with normal stem cells in terms of their unlimited capacity for self-renewal [1]. They can also divide asymmetrically to generate differentiated cancer cells which contribute to the heterogeneity of tumor. These CSCs can promote tumor progression, heterogeneity, drug resistance, recurrence, and metastasis [3–9]. On the other hand, the clonal evolution model hypothesizes that cancer originates from any cell type. These cells accumulate various mutations stepwise over time and through genetic instability acquire CSC characteristics necessary to adapt to stress and the changes of microenvironment. Thus, it is the most adaptive cells within the heterogeneous cancer cells that are responsible for tumor progression and relapses [10]. Each model has supporting evidence; however, new evidence indicates that a hybrid model combining these two hypotheses best describes tumor heterogeneity.

Cancer initiation and progression often is a result of deregulated self-renewal pathways in normal stem cells or due to carcinogenic mutations in other cell types [1]. In some cases, genetic mutations can activate self-renewal pathways and preserve the epigenetic programs that regulate asymmetric cell divisions thus producing cancer stem cells capable of generating heterogeneous cancer cells. In other cases, mutations cause inhibition of differentiation and allow cancer cells to maintain proliferative potential which avoids hierarchical organization [11]. Thus, whether a cancer more closely follows the CSC hypothesis or clonal evolution concept depends on the cell of origin (a.k.a. tumor-initiating cell) as well as the specific genetic mutations acquired and their consequences. Whichever is the dominant model, investigating the underlying mechanisms of BCSC characteristics will allow us to further understand how breast cancer cells propagate and identify new biomarkers and therapeutic targets.

### Identification of heterogeneous BCSCs

Work in hematopoietic stem cells using cell surface markers has made it possible to identify a differentiation hierarchy of cell lineage with defined functional characteristics [12]. In the mammary gland, a similar stem/progenitor/differentiated cell hierarchy has been described [13–16]. Using specific cell surface markers and fluorescence-activated cell sorting (FACS) to isolate mammary stem cells from mice, the concept of a single mammary stem cell being able to reconstitute the whole mammary gland was validated [15,16]. Subsequently, mammary progenitor cells specific for myoepithelial/basal or luminal lineage have also been characterized in mice [17–19].

To isolate and characterize BCSCs, surface markers in combination with cell sorting and assays including soft agar colony formation, mammosphere formation, epithelial-mesenchymal transition (EMT) marker expression, asymmetric cell division and mouse xenotransplantation and tumorigenesis assays are used. The first and most commonly used markers to enrich BCSCs are CD44 and CD24 [20]. Cells with a specific pattern of these markers (CD44^+^, CD24^−/low^, epithelial specific antigen (ESA)^+^) but lacking expression of specific lineage markers (Lin^−^) exhibited EMT phenotypes [21]. These cancer cells also had higher tumorigenic potential than bulk tumor cells after transplantation in nonobese diabetic/severe combined immunodeficient (NOD/SCID) mice [20,21]. In a low attachment *in vitro* culture system, these cancer cells were able to form clonal nonadherent mammospheres which were more tumorigenic than established breast cancer-derived cell lines including MCF-7 and B3R [22].

After this discovery, many efforts have been put into additional marker identifications, both on the cell surface and in the cytoplasm, to further isolate BCSCs [23–29]. For example, protein C receptor (PROCR, a.k.a. CD201) was identified using gene expression profiling of primary breast cancers [24]. CD133 was identified for BCSCs isolated from cell lines generated from Brca1^−exon11^/p53^+/−^ mouse mammary tumors [25]. The cytoplasmic marker aldehyde dehydrogenase (ALDH) was found in creased in a subpopulation of both normal and cancerous human mammary epithelial cells that exhibited stem/progenitor cell properties [23]. Some BCSCs also exhibited low proteasome activity [28]. These observations indicated that BCSCs themselves may be heterogeneous with different BCSC types present in different breast cancer subtypes. Our laboratory used a panel of known stem cell markers and found that the expression of these markers varied greatly among breast cancer cell lines and primary tumors [26]. An association between metastasis status and a high prevalence of certain markers including CD44^+^/CD24^−/low^, ESA^+^, CD133^+^, C-X-C chemokine receptor type 4 (CXCR4)^+^ and PROCR^+^ in primary tumor cells was also found. We further identified a highly malignant BCSC subpopulation from MDA-MB-231 cells with a PROCR^+^/ESA^+^ marker signature rather than the previously identified CD44^+^/CD24^−/low^ and ALDH^+^ pattern. This PROCR^+^/ESA^+^BCSC subpopulation was highly enriched with cancer stem/progenitor cell populations. Our results suggested that similar to leukemia, several stem/progenitor cell-like subpopulations can exist in breast cancer. In agreement with our study, Pece and coworkers [29] utilized the quiescent nature of normal mammary stem cells which can retain a lipophilic fluorescent dye PKH26 to further identify additional marker combinations for BCSC isolation. They found that CD24^high^/ CD49^high^/DNER (Δ-notch-like EGF repeat-containing transmembrane) ^high^, CD24^high^/CD49^high^/DLL1 (Δ-like-1)^high^, and CD49f^+^/DLL^high^/ DNER^high^ can be used as markers and provided additional evidence for the concept of BCSC heterogeneity. Whether these BCSC markers are associated with stem cell functions and characteristics, or just serve as a tool for BCSC identification needs to be further investigated.

### Regulatory mechanisms to maintain or acquire BCSC characteristics

**Figure 1:**
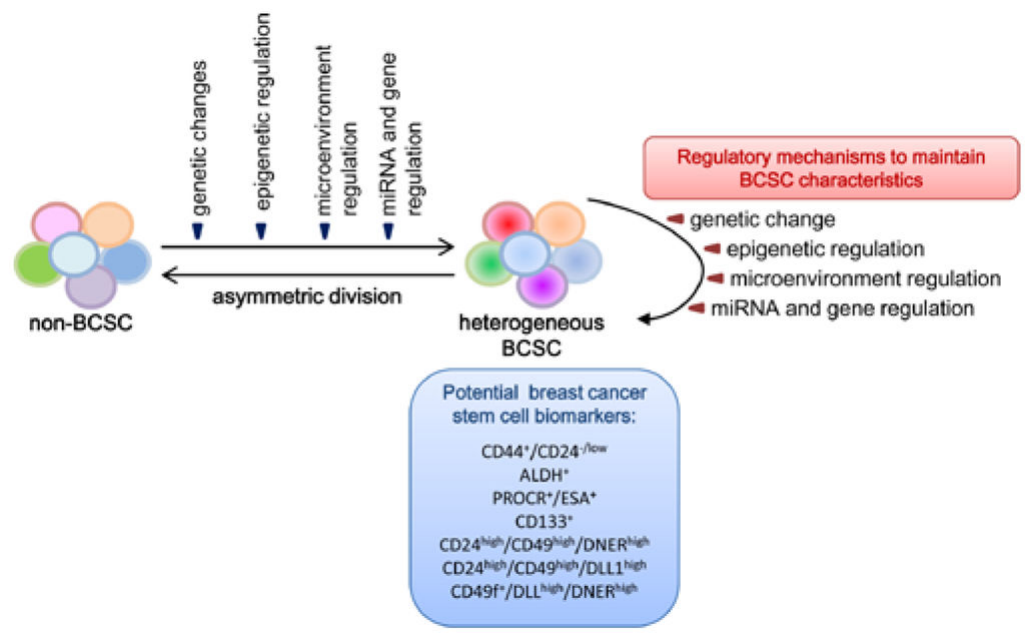
A complex multidirectional relationship between differentiated cancer cells and heterogeneous BCSCs Depending on genetic and epigenetic changes, miRNA and gene regulation or microenvironment stimuli, cancer cells are able to shift between stem-like and non-stemlike states. This plasticity contributes to the heterogeneity of BCSCs. The regulatory mechanisms underlying the shift from non-BCSCs to BCSCs also play important roles in maintaining the BCSC pool and its characteristics. The currently used cell surface markers for BCSC identification could be potentially useful clinical biomarkers.

The stem/progenitor/differentiated cell hierarchy is not a unidirectional system. It has been shown that normal mature epithelial cells can spontaneously revert to stem cells [30] suggesting a complex multidirectional relationship between stem/progenitors and differentiated cells. This plasticity also occurs in heterogeneous cancer cells. The seemingly reverse transition from cancer cell without stem-like properties to CSC has recently been described [21,31]. Thus, CSCs are not a fixed population. Depending on genetic and epigenetic changes or microenvironment stimuli, cancer cells are able to shift between stem-like and non-stem-like states [32] (Figure 1).

#### Genetic changes

Consistent with the invasive nature of CSCs, EMT is able to drive cancer cells to acquire CSC characteristics. EMT is also important in maintaining CSC subpopulations. For example, expression of SLUG/SNAI2, TWIST, CD146 and Kruppel-like factor 4 (KLF4), which promote EMT and invasion, can drive cancer cells to express the CD44^+^/CD24^−^BCSC markers and exhibit BCSC phenotypes [33–36]. Many oncogene overexpression or tumor suppressor gene mutations also contribute to this aspect. For instance, oncogene human epidermal growth factor receptor 2 (HER2) overexpression can drive cancer cells to have higher ALDH activity and BCSC characteristics [37]. Loss of tumor suppressor p53 was found to be correlated with an increase in the expression of EMT genes and stemness markers in breast cancer cells [38].

#### Epigenetic regulation

Besides genetic changes, epigenetic regulation also determines BCSC properties. Polycomb repressor proteins have been shown to play an important role in maintaining the self-renewal capability of stem cells [39–42]. Enhancer of zeste homolog 2 (EZH2), a key component of the polycomb repressor complex (PRC) 2 which mediates histone H3 methylation at lysine 27 (H3K27), has been linked to aggressive progression of breast and prostate cancers [43,44]. Recent evidence demonstrated that EZH2 epigenetically down-regulated DNA damage repair protein Rad51 leading to activated extracellular signal-regulated kinase (ERK)-β-catenin signaling which resulted in BCSC population expansion [45]. In addition, epigenetic inactivation of the tumor suppressor breast cancer 1 (BRCA1) gene due to CpG island hypermethylation in breast cancer was observed [46,47]. These BRCA1 defective breast cancers contained expanded luminal progenitor cells [48]. These findings demonstrated a BCSC regulatory mechanism linking genetic aberration, epigenetic regulation and DNA repair defects.

#### Microenvironment regulation

In addition to genetic and epigenetic regulation of the mammary stem/progenitor cells, autocrine signaling of these cells, paracrine signaling from the surrounding differentiated cells and the microenvironment also contributes to the regulation of self-renewal and differentiation [49–53]. For example, hypoxia of the tumor microenvironment has been shown to enrich CSC subpopulations and induce CSC phenotypes in many solid tumors. Anti-angiogenesis agents which cause intratumoral hypoxia resulted in an increase in BCSC prevalence via activation of the Akt-β-catenin pathway and promoted an invasive phenotype [54]. Hypoxia also caused expansion of CSC subpopulation via up-regulation of embryonic stem cell-like transcription factors including octamer-binding transcription factor 4 (OCT4), NANOG, sex determining region Y-box 2 (SOX2), KLF4 and Lin-28 [55]. In addition to hypoxia, inflammation also plays a role in regulating CSCs. For example, tumor-associated macrophages promoted CSC phenotypes including tumorigenesis and drug resistance through their downstream factors interleukin (IL)-6 and milk-fat globule-epidermal growth factor-8 which activated the signal transducer and activator of transcription 3 (STAT3) and Sonic Hedgehog pathways in CSCs [56].

Paracrine/autocrine signaling pathways between surrounding stromas/non-BCSCs and BCSCs are also important in regulating BCSC phenotypes. BCSC secreted IL-6 was found to convert breast cancer progenitor cells to a more BCSC-like phenotype through a positive feedback loop among nuclear factor of kappa light polypeptide gene enhancer in B-cells (NF-κB), Lin-28 and let-7 miRNA [57]. Recently, cancer associated fibroblasts (CAFs) were found to secret chemokine (C-C motif) ligand 2 (CCL2) and stimulate the stem cell-specific, sphere-forming phenotype and BCSC self-renewal through induction of NOTCH1 expression [58]. In addition, mesenchymal stem cells (MSCs) have been found in the tumor-associated stroma in breast cancer. Breast cancer cells stimulated MSCs to secret chemokine CCL5, which then acts in a paracrine fashion on the chemokine receptor CCR5 in cancer cells to promote their metastatic potency [59]. Also, endothelial cells contribute to the BCSC regulation by increasing the expression of metaphase cell-cycle genes in CD44^+^/CD24^−^BCSCs. These endothelial cells promoted cancer cell proliferation by secreting platelet-derived growth factor subunit B (PDGFB), which was stimulated by cancer cell-secreted vascular endothelial growth factor (VEGF), fibroblast growth factor 12 (FGF12), pleiotrophin (PTN) and neurofibromin 1 (NF1) [60].

#### micro-RNA and gene regulation

Likewise, micro-RNAs (miRNAs) contributing to the promotion or maintenance of stemness of cancer cells have also been found. In many cases, miRNAs possessing tumor suppressor functions were down-regulated and those exhibiting oncogenic functions up-regulated in BCSC subpopulations. This differential miRNA expression contributed to maintenance of the BCSC population as well as stem cell phenotypes. In mouse mammary stem/progenitor cells, both miR-205 and miR-22 were highly expressed whereas miR-93 and let-7 were down-regulated [61]. Over-expression of miR-205 increased the progenitor cell population [62]. Similarly, in human breast cancers, tumor suppressor miRNA let-7 which inhibits self-renewal capacity and promotes differentiation by repressing H-Ras and high mobility group AT-hook 2 (HMGA2)was found to be downregulated in the CD44^+^/CD24^−/low^ BCSCs [63]. Shimono and coworkers found 37 miRNAs differentially expressed between BCSCs and non-BCSCs. Among these miRNAs, miR-200c was found to inhibit BMI1 polycomb ring finger oncogene (BMI1), a regulator of stem cell self-renewal, and led to inhibition of clonal expansion of breast cancer cells *in vitro*, and strongly suppressed the tumor formation driven by BCSCs *in vivo* [64]. miR-200 has also been found to downregulate suppressor of zeste 12 homolog (Suz12), a subunit of a polycomb repressor complex, which has important roles in BCSC formation and maintenance [65]. However, the importance of other differentially expressed miRNAs in BCSCs remains to be determined.

To identify a general mechanism of promoting BCSC phenotypes, we compared the miRNA expression profile of PROCR^+^/ESA^+^BCSC subpopulation [26] with that of the CD44^+^/CD24^−/low^ BCSC subpopulations [64]. This strategy allowed us to elucidate molecular mechanisms that are either shared among different BCSCs or unique to the highly tumorigenic PROCR^+^/ESA^+^ subpopulation. We identified miR-495 as an oncogenic miRNA upregulated in both the PROCR^+^/ESA^+^ and CD44^+^/CD24^−/low^ BCSC sub-populations[66]. Using multiple cell lines expressing different stem cell markers, we found that, regardless of cellular context, miR-495 could maintain or promote colony-forming ability, promote EMT marker expression, downregulate E-cadherin and suppress DNA-damage-inducible transcript 4 (DDIT4, a.k.a. REDD1)to confer hypoxia tolerance. These observations further suggested that although heterogeneous BCSCs might have dissimilar ability in tumorigenicity, migration and invasion, and maybe associated with different subtypes of breast cancers, there may be shared regulatory pathways that maintain BCSC phenotypes.

### Targeting breast cancer stem cells

Biopsies from patients treated with neoadjuvant chemotherapy showed enrichment of BCSCs [63,67]. Similarly, enrichment of BCSCs was also found in tumors spontaneously developed or engrafted in animals receiving chemotherapy or radiation treatment, respectively [68,69]. The conversion of non-BCSCs to BCSCs was more prevalent after endocrine therapy [27,70–72]. Thus, the inherent resistance of BCSCs to chemo- and radiotherapy could be the major source of disease recurrence.

Many efforts have been put into finding treatments to eliminate BCSCs. Two major approaches have been used: 1, drug screening to identify chemicals selectively toxic to BCSCs and 2, targeting specific regulatory factors involved in regulating BCSC phenotypes. For example, Gupta and colleagues [73] utilized a chemical screen to identify drugs with selective toxicity for BCSCs and found that salinomycin was able to reduce the prevalence of CD44^+^/CD24^−^ BCSCs compared to paclitaxel, a commonly used breast cancer chemotherapeutic agent. More recently, a combinatorial therapy with conventional chemotherapeutic agent and the anti-diabetic drug metformin has been shown to efficiently eliminate drug-resistant CD44^+^/CD24^−^ cancer stem/progenitor cells in breast cancers [74, 75].

Investigation of differential gene expression between CSCs and non-CSCs makes it possible to find potential therapeutic targets. For example, gene expression profiling indicated that CXCR1, a cytokine IL-8 receptor, was up-regulated in ALDH^+^ BCSCs. Moreover, recombinant IL-8 treatment increased the BCSC subpopulation and promoted its invasion ability [76]. Wicha’s group used either a CXCR1-specific blocking antibody or repertaxin, a small-molecule CXCR1 inhibitor, to block CXCR1 and found that the ALDH^+^BCSC subpopulation was selectively depleted and this was mediated by the FAK/AKT/FOXO3A pathway [77]. Polyak’s group identified 15 genes required for cell growth or proliferation in CD44^+^CD24^−^BCSCs in a large-scale loss-of-function screen. They found that inhibition of several of these such as IL6, prostaglandin I2 synthase (PTGIS), hyaluronan synthase 1 (HAS1), CXCL3, and 6-phosphofructo-2-kinase/fructose-2,6-biphosphatase 3 (PFKFB3) reduced STAT3 activation suggesting that the IL-6/JAK2/STAT3 pathway was preferentially active in CD44^+^CD24^−^BCSCs. Indeed, inhibition of JAK2 was able to decrease theCD44^+^CD24^−^BCSC number and block growth of xenografts [78]. More recently, Hung’s group showed that BIKDD, a constitutively active mutant form of proapoptotic gene, BIK, significantly reduced CD44^+^/ CD24^−^ BCSCs through co-antagonism of its binding partners B-cell CLL/lymphoma 2 (Bcl-2), Bcl-xL, and myeloid cell leukemia sequence 1 (Mcl-1), suggesting a potential therapeutic strategy targeting BCSCs [79].

Although these approaches are worthwhile to develop targeted therapies, there are some limitations. As described above, BCSCs are heterogeneous and different BCSCs may be associated with different subtypes of breast cancers. Most of the studies only focused on one specific type of BCSCs, i.e., CD44^+^/CD24^−^ subpopulation. Whether the potential therapeutic targets possess the same efficacy among different types of BCSCs remains to be determined. Thus, identification of the shared mechanism in different types of BCSCs, especially the regulation in self-renewal and asymmetric division, is important for treatment design. Moreover, whether therapies deplete BCSC by inducing cell death or promoting differentiation can be important in determining the treatment efficacy. As discussed above, BCSCs can arise from more differentiated cancer cells [30] to maintain the dynamic equilibrium between BCSCs and non-BCSCs [80]. The plasticity and adaptability of aggressive cancer cells with BCSC phenotypes, in combination with genomic instability, may reduce the treatment efficacy. If the targeted therapies deplete BCSCs by causing BCSC specific cell death, this may promote the transient-amplifying cancer cells to convert to BCSC-like cells. Thus, therapies which induce BCSC differentiation or target the pathway regulating the plasticity may be more favorable.

While designing targeted therapies based on BCSC phenotypes and the regulatory pathways, one should also take the tumor microenvironment into account. Our laboratory, along with others, has shown that the cancer associated fibroblasts can promote tumorigenesis as well as BCSC phenotypes via paracrine signaling [58,81,82]. Thus, a combination of treatment skilling bulk cancer cells, targeting BCSCs and their surrounding microenvironment may be the most efficient way to treat cancer.

## Final Remark

In this exciting field, the number of cell surface markers for identifying BCSCs are growing exponentially. The ultimate goal is to use the best reliable BCSC markers as biomarkers for clinical use (Figure 1). However, whether the current BCSC markers can be used as biomarkers remains to be determined. By definition, the best biomarker is a unique molecular signature that can be unambiguously correlated to biological events in order to validate novel drug targets, predict drug response, and eventually help therapeutic decision making [83]. Thus, a BCSC biomarker will need to be reproducible and measurable in clinical samples to detect the BCSC subpopulation which determines outcomes from cancer or responses to treatment [84,85]. As discussed above, the current system to identify, isolate and study relatively rare BCSCs from primary specimens remain difficult for clinical practice. Unless a better system can be established to perform large cohort studies for profiling the heterogeneous BCSCs and their corresponding breast cancer types, the currently identified BCSC markers may not meet the need for establishment of clinically relevant biomarkers. Thus, continuing effort in identifying functional markers involved in regulating BCSC phenotypes and improving BCSC identification methodology is also essential in moving our new knowledge of BCSCs from laboratory to clinical practice.

Specifically targeting BCSCs for therapeutic treatment relies on characterizing BCSCs and their microenvironment. Continuing efforts in identifying heterogeneous BCSCs and their genetic/epigenetic similarities and differences are needed. To achieve this, the limitations in current methods and techniques for BCSC identification have to be conquered. One key limitation is that there is yet no suitable system to maintain primary BCSCs in long term culture for detailed mechanistic studies. Limited availability of primary specimens, in addition to the low prevalence of BCSCs, makes many methods not easily applicable in routine practice for large cohort studies. Most experiments must instead rely on a relatively small number of immortalized human breast cancer cell lines. As new evidence has indicated the importance of the BCSC niche in BCSC regulation, identification and characterization, the stromal or endothelial cells surrounding BCSC may be useful for improving long term culture for mechanistic studies.
